# Supplementary material for: Transiently Transfected Mammalian Cell Cultures: An Adaptable and Effective Platform for Virus-like Particle-Based Vaccines against Foot-and-Mouth Disease Virus
Source: Viruses. 2022 May 7;14(5):989. doi: 10.3390/v14050989 (PMC9147724; doi:10.3390/v14050989)
Supplement: Supplementary file 1 [file viruses-14-00989-s001.zip › viruses-1670096-supplementary.pdf]

Supplementary Figure

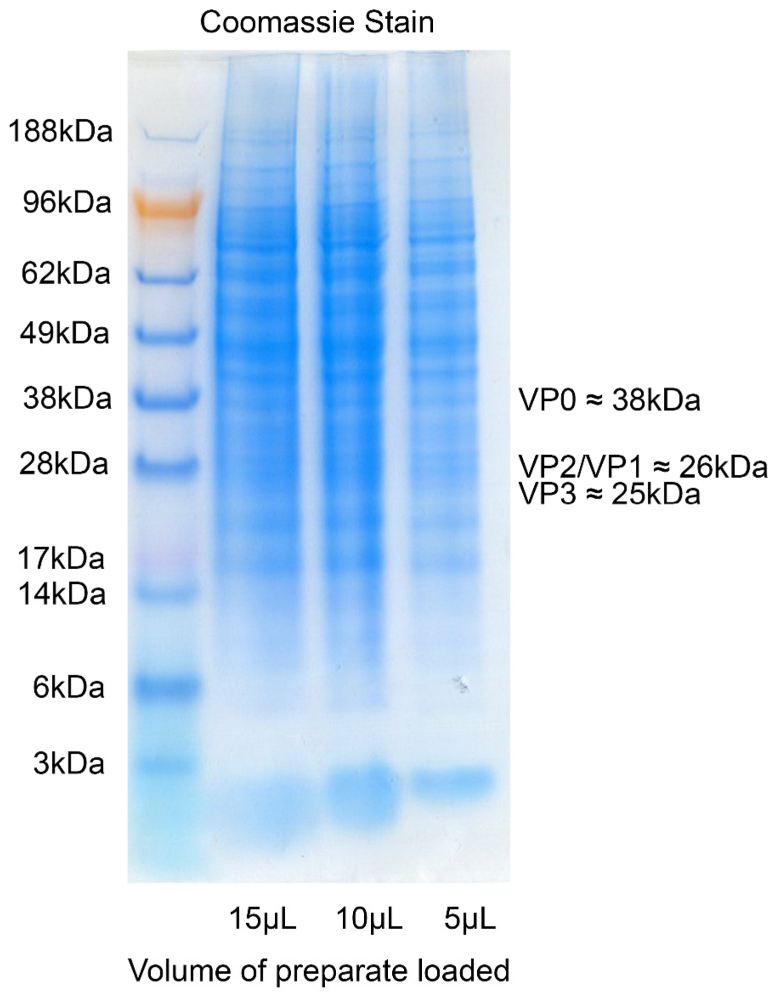

SDS-PAGE gel with Coomassie stain of O1M VLP vaccine preparate. Different volumes of the preparate were loaded onto the gel to better allow for visualization of protein components. Additional bands not associated with FMDV antigen are a result of the methodology used in this manuscript, as lysis of the cells results in carryover of proteins from the production cell line into the preparate.
